# Working conditions and stressors data during Covid-19 and mental well-being in Iranian healthcare workers

**DOI:** 10.1016/j.dib.2022.108551

**Published:** 2022-08-23

**Authors:** Marzieh Belji Kangarlou, Farin Fatemi, Alireza Dehdashti, Fatemeh Paknazar

**Affiliations:** a Student Research Committee, Semnan University of Medical Sciences, Semnan, Iran; b Research Center of Health Sciences and Technologies, Semnan University of Medical Sciences, Semnan, Iran; c Social Determinants of Health Research Center, Semnan University of Medical Sciences, Semnan, Iran

**Keywords:** Data, Occupational risk, Stress, Burnout, Health care settings

## Abstract

The current Covid-19 pandemic has affected the physical and mental stressors of hospital-based healthcare workers, but the extent of such effects are required to be quantified. This survey looked at data on nurses’ perception across teaching hospitals to assess the impacts of Covid-19 on working conditions, exposure to stressors, and mental health symptoms. We implemented a population survey with a cross-sectional design in teaching hospitals affiliated with Medical Sciences Universities in Iran from April to November 2021. Participants were about 1200 health care workers, including hospital nursing staff, assistants, and technicians. Final data were assembled from 831 hospital nurses across surgery, dialysis, intensive care, emergency care, cardiac care, internal medicine, gynecology, and pediatric wards. Self-reported data were collected directly from survey participants. We collected information on variables including gender, marital status, employment status, occupational health training, evaluation of work environment stressors, fear of Covid-19, and occupational burnout constructs, specifically reflecting emotional exhaustion, depersonalization, and personal accomplishment. Focus groups of faculties evaluated and edited items to test the content wording and to define the content that are valid measures of the variables. The questionnaires were assessed for their reliability. Manual data entries were double-checked for errors. Data were recorded and categorized consistently to ensure the replicability of the data in the future. Statistical descriptive and analytical analyses were performed on the data. Data reported on the frequencies and mean values of responses and the variations of mental health in terms of worktime schedules. Chi- square, ANOVA, and correlation analyses determined relations between variables. The compiled data shed light on the exposure and response to physical and psychosocial factors and mental health symptoms among nurses during the pandemic. The data files detailed in this article can be further reused to inform workplace determinants of health in hospital settings. The obtained scores and existing dataset on mental health outcomes can help future studies to consider resilience strategies that should be provided among nurses.

**Specifications Table**

**Table:** 

Subject	Health and medical sciences Occupational Health Psychology
Specific subject area	Occupational stressors contributed to mental health among hospital nurses
Type of data	Table chart
How the data were acquired	Data were assembled through standard instrument consisted of four parts. The first part asked questions for obtaining demographic and work characteristics including nurses’ gender, age, marital status, the place of work, the length of nursing work, shift work schedule, education level, and employment status. The second part applied the Iranian version of the Burnout Inventory for detecting and assessing the severity of burnout syndrome. This tool assessed three domains of emotional exhaustion (9 items), depersonalization (8 items), and personal accomplishment (5 items). The third part of the questionnaire collected information on the perceived nursing workload by Task Load Index (TLX). The fourth part of the questionnaire measured fear perceived by nurses during the spread of SARS-COV-2. Questionnaires were distributed in person among hospital nurses from April to November 2021. We asked participants to rate their responses to burnout symptoms, workload, and feeling of fear in Likert scales. We collected data anonymously and coded them to ensure confidentiality. the individuals involved in collecting data were instructed to provide target population with the objectives of research and record and categorize data in a consistent way so that the data can be replicated in the future. Manual data entries were double-checked for errors. To achieve the objectives, the research team defined the content that are valid measures of the variables such as burnout, task load, and feeling of fear. The survey questions were evaluated and edited through focus groups to test content wording. The questionnaires were assessed for their validity and reliability.
Data format	Raw analyzed
Description of data collection	The inclusion criteria were having a nursing work experience of at least two years and signing an informed consent form. Nurses contracted with Covid-19 or identified with previous psychiatric or mental disorders or unwilling to respond or partially filled out questionnaires were removed. Of all distributed in-person questionnaires among a target population of about 1200 hospital nurses, data were assembled from 831 nurses completed the questionnaire.
Data source location	*• Institution: Semnan university of Medical sciences:* *• City/Town/Region: Semnan* *• Country: Iran*
Data accessibility	With the article Repository name: Mendeley Data Direct URL to data: https://data.mendeley.com/datasets/ccdppxc6pb/2 Digital Object Identifier DOI:10.17632/ccdppxc6pb.2
Related research article	Belji Kangarlou, M.; Fatemi, F.; Paknazar, F.; Dehdashti, A. Occupational Burnout Symptoms and Its Relationship With Workload and Fear of the SARS-CoV-2 Pandemic Among Hospital Nurses. Front. Public Heal. 2022, 10. https://doi.org/10.3389/fpubh.2022.852629

## Value of the Data

• The data for assessing occupational stressors and their relationships with burnout is essential to manage workplace health services and mitigate potential consequences.
• Collecting data concerning nurses’ working environment and conditions is necessary because excessive exposure to work-related and individual risk factors has implications for the health and profession of nurses.
• This study compiled data to shed light on the association between working conditions and burnout symptoms among nurses during the pandemic.
• The current data will allow workplace health providers to meet emergent needs for health-related information to respond to pressing occupational burnout issue.
• The data detailed in this article can be further reused to inform workplace determinants of health during the pandemic in hospital settings.
• These data can be utilized for proposing and assessing preventive measures in future studies for improving nurses’ safety and health at work during the pandemic crisis in hospital settings.
• The dataset during pandemic can be reanalyzed for later comparisons to nurses’ perception in post pandemic era.
• The obtained scores and existing dataset on mental health outcomes can help future studies to consider resilience strategies that should be provided among nurses.

## 1 Background

Literature reviews reported health care workers across the globe working continually under high-stress Covid-19 conditions have reported fatigue, distress, anxiety, and depression [1], [2], [3]. A study indicated higher prevalence of mental health problems among Iranian health care workers during the Covid-19 pandemic [4]. The Covid-19 pandemic has affected the capacities of Iranian public healthcare systems to provide health services and protect healthcare workers [5]. The pandemic has also affected working conditions and the physical and mental stressors of hospital-based healthcare workers in Iran, but the extent of such effect are required to be quantified [6]. While providing dataset regrading nurses’ work environment conditions and their mental health is crucial in a public health crisis, very few support references are presented. In addition, healthcare workers have an irreplaceable role in the global-scale information process of knowledge management for dealing with Covid-19. When they experienced stress and burnout, this vital link was weakened and impacted the whole public health system [7]. The rational of the current dataset's values based on three aspects. First, the availability and sharing the dataset related to the potential predictors of health workers' mental well-being during the Covid-19 pandemic can help promote innovative research and eventually the effectiveness and efficiency in health system management [6]. Second, data sharing offers valuable resources for scientific research and evidence-based policymaking, especially in developing countries, where scientific investments are limited. Third, data sharing is a practice that helps improve research integrity and transparency through enabling reproduction and peers' validation of the study's findings [7].

## 2 Data Description

This paper presented data on exposure and responses to stressors and occupational determinants of mental health symptoms that hospital nurses experienced in Iran during the pandemic. The authors of this dataset published an original research article included data and methodology to highlight health risks in hospital work environment [8]. Data were weighted to represent estimates of hospital nurses from surgery, dialysis, intensive care, emergency care, cardiac care, internal medicine, gynecology, and pediatric wards. We collected data from 831 nurses employed at teaching hospitals who completed and returned the questionnaires. The survey's raw data are provided in the repository [9].

Table 1 presents the overall population, participants, and response rates for each hospital setting. The participants included nurses between 23 and 49 years old (M = 34.7 years, SD = 0.7). Most nurses who participated in the study were female, 602(72.5%) versus male, 229 (27.5%). Length of service as a nurse varied between 2 and 29 years (M = 11.2, SD = 0.6). Most male and female nurses graduated from higher education institutions (93.1%). Over half of the nurses evaluated safety and health in their work settings as unacceptable (58%). A higher proportion of nurses reported having training for safe work procedures (84%) in the hospital environment. About one-fifth of the hospital nurses worked overtime during the pandemic.

**Table 1:** Data on target population, respondents and response rates of nurses in each hospital, April to November 2021.

Settings	Target population *n*	Respondents *n*	Response rate %
Hospital A	265	189	71.3
Hospital B	247	171	69.2
Hospital C	268	174	64.9
Hospital D	212	134	63.2
Hospital E	216	163	75.4
Total	1208	831	68.8

Table 2 shows the internal consistencies of the questionnaires. Cronbach's alpha factors estimated above 0.7 for the workload, fear of covid-19, and burnout symptoms constructs.

**Table 2:** Data on the internal consistencies of the constructs.

Subscales	Number of items	Cronbach's factors	Internal consistency
Task load index	4	0.71	Acceptable
Fear of Covid-19	4	0.84	Good
Emotional exhaustion	9	0.84	Good
Depersonalization	8	0.76	Acceptable
Personal achievement	5	0.79	Acceptable

**Chart 1:**
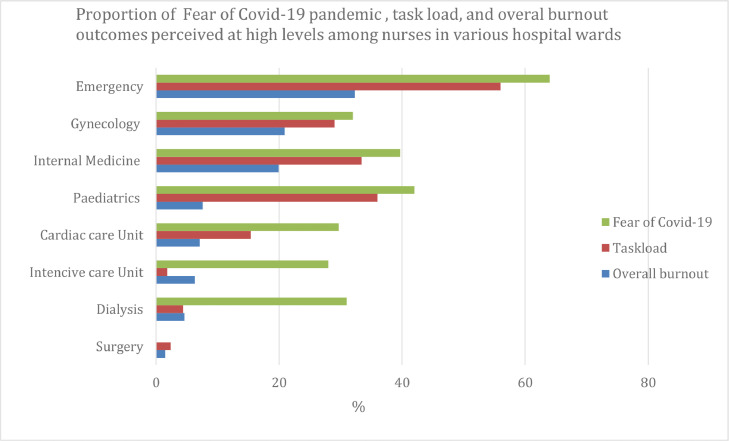
Proportion of perceived fear of Covid-19, task load, and overall burnout among nurses in various hospital wards, April to November 2021. Source

Burnout is determined as having a high level in each of the burnout domains of the instrument. The data indicated that about one in three (33%) of nurses screened for a high level of burnout symptoms. Among hospital nurses, one-third (31%) perceived workload at moderate or high levels. Most participating nurses (83%) rated their feeling of fear due to Covid-19 as high. In Chart 1, we created a histogram of the data on overall burnout, task load, and fear of Covid-19 in various hospital wards. Nurses who worked in the emergency experienced higher proportion burnout symptoms.

The scores for emotional exhaustion was (M = 37.09, SD = 0.8), depersonalization (M = 24.9, SD = 0.3), and personal accomplishment (mean = 18.9, SD = ± 0.3). The number of responses to each item of the burnout construct is presented in Table 3.

**Table 3:** Responses to mental health items defining occupational burnout domains and symptoms as perceived by hospital nurses from April to November 2021 (0 = Never, 1 = At least a few times a year, 2 = At least once a month, 3 = Several times a month, 4 = Once a week, 5 = Several times a week, 6 = Every day).

	Likert scales						
Questions	0	1	2	3	4	5	6
	**Frequency of responses (*n*)**						

*Emotional exhaustion*							

I feel emotionally exhausted because of my work	25	19	61	56	178	358	134
I feel worn out at the end of a working day	18	32	22	98	223	281	157
I feel tired as soon as I get up in the morning and see a new working day stretched out in front of me	26	34	81	79	315	185	111
Working with people the whole day is stressful for me	129	99	84	87	76	174	182
I feel as if I'm at my wits ‘end	28	106	117	185	176	121	98
I feel frustrated by my work	24	108	159	181	137	129	93
I get the feeling that I work too hard	12	43	112	149	194	216	105
I feel burned out because of my work	29	95	146	118	138	208	97
8. Being in direct contact with people at work is too stressful							

*Depersonalization*							

I have become more callous to people since I have started doing this job	37	143	115	129	107	106	94
I'm afraid that my work makes me emotionally harder	24	106	139	168	179	114	101
I get the feeling that I treat some clients/colleagues impersonally, as if they were object	103	104	193	121	98	129	83
I have the feeling that my colleagues blame me for some of their problems	67	89	112	175	138	153	97
I'm not really interested in what is going on with many of my colleagues	18	32	165	113	129	270	104

*Professional achievement*							

I can easily understand the actions of my colleagues/supervisors	137	56	102	142	295	21	78
I deal with other people's problems successfully	41	124	102	175	207	79	103
I feel that I influence other people positively through my work	81	115	97	149	137	150	102
I feel full of energy	172	132	107	22	147	148	103
I find it easy to build a relaxed atmosphere in my working environment	18	128	96	184	194	126	85
I feel stimulated when I been working closely with my colleagues	124	132	107	178	195	16	79
I have achieved many rewarding objectives in my work	139	86	31	182	201	86	106
In my work I am very relaxed when dealing with emotional problems	143	62	140	183	176	17	110

The mean scores assigned to each item measured burnout structure by the length of work shifts are presented in Table 4. The Statistical *P*-values of data at or less than 0.05 indicate differences in burnout symptoms between nurses who work at regular and extended working hours.

**Table 4:** Nurses’ perceptions of burnout symptoms (data collected from April to November 2021) Values of responses based on scale options from never=0 to everyday=6.

	Regular working hours ≤8		Extended working hours ≥8			
*Items*	Mean	SD	Mean	SD	X^2^	P
*Emotional exhaustion*						

I feel emotionally exhausted because of my work	5.08	1.88	5.78	1.43	14.6	0.002
I feel worn out at the end of a working day	4.44	2.02	5.79	1.05	18.1	0.001
I feel tired as soon as I get up in the morning and see a new working day stretched out in front of me	4.79	2.17	5.10	1.83	16.0	0.018
Working with people the whole day is stressful for me	5.31	1.47	5.19	2.03	12.5	0.104
I feel as if I'm at my wits ‘end	3.09	1.54	3.97	1.98	18.4	0.087
I feel frustrated by my work	5.05	1.24	5.48	1.67	27.0	0.031
I get the feeling that I work too hard	4.33	1.55	4.59	1.37	13.7	0.170
I feel burned out because of my work	4.58	1.88	4.78	1.06	14.7	0.540
Being in direct contact with people at work is too stressful	5.14	1.65	5.19	1.63	18.3	0.710

*Depersonalization*						

I have become more callous to people since I have started doing this job	3.85	1.23	4.76	2.07	19.5	0.173
I'm afraid that my work makes me emotionally harder?	4.36	1.91	4.21	1.58	21.0	0.204
I get the feeling that I treat some clients/colleagues impersonally, as if they were object	4.88	1.71	4.93	1.34	17.0	0.301
I have the feeling that my colleagues blame me for some of their problems	2.90	1.51	3.12	1.04	13.1	0.162
I'm not really interested in what is going on with many of my colleagues	3.23	1.60	4.05	1.29	18.4	0.091

*Professional achievement*						

I can easily understand the actions of my colleagues/supervisors	2.63	1.38	1.69	0.84	10.6	0.024
I deal with other people's problems successfully	2.82	1.77	1.73	0.73	19.6	0.002
I feel that I influence other people positively through my work	1.81	0.56	1.50	0.79	11.7	0.002
I feel full of energy	1.80	1.67	1.26	0.95	26.7	0.001
I find it easy to build a relaxed atmosphere in my working environment	1.40	0.87	1.50	0.92	18.9	0.025
I feel stimulated when I been working closely with my colleagues	2.90	1.17	1,75	1.05	11.6	0.005
I have achieved many rewarding objectives in my work	1.89	0.86	1.72	1.38	17.6	0.041
In my work I am very relaxed when dealing with emotional problems	1.54	0.76	1.64	0.56	16.3	0.160

As detailed in Table 5, comparisons of the participant groups perceived “no or low” burnout with those of “moderate or high” burnout indicated significant differences in terms of the years of employment in the nursing profession (*p* = 0.006). Furthermore, a significant difference was obtained between two groups of burnout levels regarding the average number of hours nurses worked per day in the hospital settings (*p* = 0.049). The means of participant's age did not differ significantly among the various burnout levels.

**Table 5:** Data analysis of variances comparing the means of two groups of hospital nurses based on burnout level during Covid-19.

	Nurses perceived no or low burnout		Nurses perceived moderate to high burnout			
Variables	Mean	SD	Mean	SD	F	*P*-value
Number of years employed as a nurse	6.39	3.51	16.91	5.13	22.41	0.006
Average number of hours worked per day	7.48	1.27	10.67	2.53	5.04	0.049
Age	27.80	6.18	34.15	7.82	17.3	0.071

Table 6 shows the results of data analysis for the correlation between variables. A Pearson correlation coefficient was calculated for the relationship between participants’ feelings concerning fear of Covid-19 and burnout. A positive correlation was found (r (829) = 0.604, *p* < 0.001), indicating a significant reliable relationship between the two variables. Nurses having more feelings of fear toward Covid-19 tend to experience burnout more. Additionally, a significant strong relationship was obtained between burnout and task load (r (829) = 0.723, *p* < 0.001).

**Table 6:** Data analysis related to association between burnout with fear of Covid-19 and task load (April to November 2021).

		Burnout symptoms
Fear of Covid-19	Correlation	0.604
	Sig. (2-tailed)	0.006

Task load	Correlation	0.723
	Sig. (2-tailed)	0.004

Correlation is significant at the 0.05 level (2-tailed).

## 3 Experimental Design, Materials and Methods

### 3.1 Survey Design and Data Source

This survey was a cross-sectional population-based survey. We collected data between April 11 and December 1, 2021. Data for this analysis were from the target population of about 1200 healthcare workers, including hospital nursing staff, assistants, and technicians working in different medical units of five teaching hospitals affiliated with Medical Sciences Universities in Iran. Participants engaged in emergency care, intensive care, surgery, dialysis, cardiac care, internal medicine, gynecology, and pediatrics. Health care workers who started their employment in April 2019 were eligible for the survey. We excluded health care workers who experienced mental disorders before the Covid-19 onset, were infected with Covid-19, were unwilling to respond, or partially filled out questionnaires. Self-reported data were collected directly from survey participants. As a result, we removed 377 (31.2%) nurses from the survey, finally assembled and analyzed information from the total survey sample of 831 nurses who satisfied inclusion criteria and returned the completed questionnaires.

### 3.2 Data Instrument Design and Measures

The survey applied a standard instrument consisting of four parts to assemble data. We compiled the questionnaire content in Persian language and in consultation with the Occupational Health and Biostatistics Department of Medical Sciences University. Focus groups evaluated and edited the survey questions to test content wording in Persian.

Individual and work characteristics included age, sex, marital status, education level, type of tasks, work schedule, type of employment, and years of nursing experience.

The Persian version of the Burnout Inventory was used to obtain data on mental health symptoms. The inventory consists of 22 Likert scale items with subscales measuring emotional fatigue, feelings of negativism related to the job, and low professional efficacy [10]. The participants indicated their level of agreement with statements on a 7-point scale ranging from “never” to “daily”. The cut-off points of the burnout levels were: “18=low”, “19-26=moderate” and “ ≥27= high” for emotional exhaustion, “≤5= low”, “6-9 moderate”, and “≥10= high” for depersonalization, and “≥40= low”, “34-39=moderate”, and “≤33=high” for personal accomplishment. The overall burnout would be evaluated at a high level if each of the three subscales of the burnout symptoms scored high.

Task Load Index (TLX) determined data on the perceived nursing workload (TLX) [11]. Originally, the TLX measures the work demands of six subscales. A previous study suggested that two subscales namely performance and frustration are significantly correlated to emotional fatigue and feelings of negativism [12]. Thus, these items were excluded, and the workload was assessed by asking respondents concerning their perceived physical, emotional, temporal and effort demands of their tasks. Data were stratified at ‘low’, ‘medium’ and ‘high’ levels.

A questionnaire was used to obtain data on the feeling of fear perceived by nurses during the spread of Covid-19 pandemic [13]. The scale consisted of four questions including “I am feared of infection with coronavirus”, “I am scare about taking coronavirus to home”, I am afraid for the future”, “I feel fear of death from Covid-19”. Data were obtained according to a four-point Likert scale (“no” to “high” level of scare). The scores ranged from 4 to 16. The final fear of pandemic scores classified as follows: 8 or less (insignificant), 9–12 (moderate), 13–16 (high).

For more details on the instrument, see materials in the repository addressed under Data availability statement [9].

### 3.3 Survey Implementation

After we obtained ethics approval and hospital permission for the survey, nurses were invited to participate through workplace well-being units. We conducted a pilot data collection before the main survey to test the nurses’ reactions and the duration needed to fill the designed parts of the instrument. Self- administered questionnaires were distributed in person among hospital nurses. Investigators informed participants about the survey and that it would take twenty minutes to complete the questionnaire. Before completing the questionnaire, respondents were assured that their information would be kept confidential and signed an informed consent. We asked participants to answer demographic questions and rate their responses to burnout symptoms, workload, and feelings of fear on Likert scales. We collected data anonymously and coded them to ensure confidentiality. The investigators involved in collecting data were trained to record and categorize data in consistently way so that the data could be replicated in the future. Manual data entries were double-checked for errors. To achieve the objectives, the research team defined the contents that were valid measures of the variables such as burnout, task load, and feeling of fear. The questionnaires were assessed for their validity and reliability.

### 3.4 Data Validation

The collected questionnaires were examined for duplicate records before processing data, as participants might be selected from multiple departments to avoid analysis with incorrect data.

Error detection of invalid and missing values was conducted on the collected data for individual and work variables and items of measured structures. We cleaned raw data by removing unwanted observations of nurses with less than two years of work experience.

In questionnaire data, the structures were measured without item mean imputation to avoid decreased variability in item scores. As correlation analysis was applied on the data, no person mean imputation was used to prevent bias. Items that had missing values were dropped, and participants with fully observed data were used in the analysis to ensure unbiased estimates.

### 3.5 Data Statistical Analyses

The assembled data were assessed using descriptive and analytical statistics. Descriptive statistics applied to summarize and calculate the percentage of stress and health outcomes during the pandemic. We calculated the frequencies of responses to each item, measuring the burnout structure to produce the percentage of participants who experienced burnout symptoms. Cross-tabulation was obtained to examine the mean values of rated responses to mental health items by worktime schedule. The data on the working hours of hospital nurses were included as a categorical predictor to analyze the variations of burnout symptoms between nurses with usual shift length and overtime work. For the Individual item of the questionnaire, the Mean values of nurses’ responses and standard deviation were calculated. Chi-square tests were performed to determine the difference between the two groups of categorical variables related to the perception of items measuring burnout. We applied the ANOVA test to determine the statistical significance of differences between nurses with no or low burnout and moderate or high burnout by the mean scores of years employed as a nurse, the number of hours worked per day, and age. Pearson correlation coefficients were calculated to examine the relationship between participants’ burnout and fear of Covid-19 and task load. A p-value of 0.05 or less was judged significant. Analyses were conducted using IBM-SPSS version 22 statistics software.

### 3.6 Dataset's Limitations

This data survey has the following limitations:

• We did not include all variables on psychosocial and organizational factors that could have helped address mental health among healthcare workers. Future research should revise the method for gathering such data.
• While this cross-sectional survey indicate differences, it cannot determine cause and effect relationships.
• The self-reported approach in collecting data on work demands and mental health status may have caused health care workers to perceive more communally acceptable responses rather than being truthful. Thus, respondents might not assess themselves accurately during the stressful covid-19 pandemic.
• Although our study included a representative sample of hospital nurses, collected data from the target population of hospital settings that did not evenly distribute in all provinces across the country might limit the generalizability of the findings to the population at large.
• This survey collected data voluntarily and thus subject to biases, and caution must be undertaken in interpreting these data.

## 4 Ethics Statements

The data survey based on a research proposal (A-10-88-22). The research protocol was reviewed and approved by the Institutional Review Board of Medical Sciences University (IR.SEMUMS.REC.1400.176). We conducted the research process in compliance with ethical procedures for a survey. We obtained required permission and support letter for implementing data collection from hospital administration. Participants were informed about the purpose of study and the topics covered in the survey and ensured about the confidentiality of their personal identifying information. Respond to the survey was voluntary and participants were asked to sign an informed consent form before completing the questionnaires.

## CRediT authorship contribution statement

**Marzieh Belji Kangarlou:** Investigation, Data curation, Writing – review & editing, Writing – original draft. **Farin Fatemi:** Formal analysis, Writing – review & editing. **Alireza Dehdashti:** Methodology, Resources, Data curation, Formal analysis, Writing – review & editing. **Fatemeh Paknazar:** Conceptualization, Data curation, Writing – review & editing, Formal analysis.

## Declaration of Competing Interest

The authors declare that they have no known competing financial interests or personal relationships that could have appeared to influence the work reported in this paper.

## Data Availability

Dataset on Working conditions and stressors and perceived mental health among Iranian healthcare workers (Original data) (Mendeley Data).
